# The Accelerated Phase of Chediak-Higashi Syndrome: The Importance of Hematological Evaluation

**DOI:** 10.4274/tjh.2012.0027

**Published:** 2013-03-05

**Authors:** Shreekant Bharti, Prateek Bhatia, Deepak Bansal, Neelam Varma

**Affiliations:** 1 Post Graduate Institute of Medical Education and Research, Department of Hematology, Chandigarh, India; 2 Post Graduate Institute of Medical Education and Research, Department of Pediatric (Hematology), Chandigarh, India

**Keywords:** Accelerated phase, Chediak Higashi, Hematology

**To the Editor**

Chediak-Higashi syndrome is a rare autosomal recessive disease that was first described in 1943 by Bequez-Cesar in 3 siblings that bore the primary clinical features. In 1952 Chediak (a Cuban hematologist) and in 1954 Higashi (a Japanese pediatrician) described a series of cases characterized by misdistribution of myeloperoxidase in the patients’ neutrophilic granules [1,2]. 

Mean age of onset is 5.85 years; however, most patients die before age 10 years. In patients that do survive beyond childhood the neurologic problems persist and/or increase in magnitude [3].

Most cases are diagnosed clinically based on partial albinism and recurrent pyogenic infections. Both of our patients had hypopigmentation of the skin with patchy grey hairs, mild coagulation defects identified via the presence of petechial rashes on the skin, and a history of recurrent infections since birth. 

The characteristic hematological finding is massive lysosomal inclusions in all white cells, formed via a combined process of fusion, cytoplasmic injury, and phagocytosis due to a microtubular defect [3]. These granules exhibit both azurophilic and specific granular markers (Figure 1), and are strongly myeloperoxidase positive. Approximately 85% of cases develop a fatal accelerated phase characterized by pancytopenia, hemophagocytosis, and marked infiltration of organs by lymphocytes, leading to multi-organ dysfunction [4]. Herein we present 2 cases in the accelerated phase of Chediak-Higashi syndrome. Both were born to apparently healthy non-consanguineous parents. 

Case 1 was a 2.5-year-old female that presented with a 6-month history of abdominal distension and moderate pallor, and a 5-d history of cough. General physical examination showed that she was thin built, and had normal facies with patchy grey hair and hypopigmentation of the skin. Systemic examination showed hepatosplenomegaly; the liver was 6 cm below the right costal margin and the spleen was 13 cm below the left costal margin. The cardiovascular and respiratory systems were normal. She also had bilateral cervical lymphadenopathy. Case 2 was a 3-year-old female that presented with a 3-month history of fever, and a 1-month history of abdominal distension, itching over the body, and moderate pallor. Physical examination showed patchy gray hair and generalized albinism with petechial spots on the thighs and abdomen. The patient weighed 7.6 kg, was of average build with normal facies and significant hepatosplenomegaly; the liver was 4 cm below the right costal margin and the spleen was 7 cm below the left costal margin. 

Both patients underwent bone marrow aspiration due to peripheral blood pancytopenia and organomegaly. Hemogram findings in cases 1 and 2 were, respectively, as follows: Hb: 4.2 g/dL and 3.9 g/dL; WBC: 4 × 109/L and 3 × 109/L; platelet count: 8 × 109/L and 6 × 109/L. Bone marrow aspiration findings in both cases are shown in the Table 1. Both patients had hematological findings characteristic of the accelerated phase of Chediak-Higashi syndrome-peripheral blood pancytopenia and marked hemophagocytosis (Figure 2) on bone marrow aspirate, along with lymphocyte infiltration on trephine biopsy section. Both cases were negative for HIV, CMV, and EBV serology. 

The genetic hallmark of Chediak-Higashi syndrome is mutations in the CHS1/LYST gene. The gene product is known to regulate lysosomal organelle function and size. The mutations could not be characterized in the presented cases because the DNA material for analysis was insufficient in one case and the other died due to the fulminant accelerated phase before hematological diagnosis was established. It is well known that Chediak-Higashi syndrome patients with deletions in the LYST gene usually present with a fulminant accelerated phase early in life, whereas those with missense mutations have a relatively better prognosis characterized by the absence of an accelerated phase and no neurological involvement [5]. 

The hematological findings of pancytopenia and hemophagocytosis suggest a possible deletion in the LYST gene in both of the presented cases, but ethnicity data and complete DNA analysis are needed to further substantiate the findings; nonetheless, both cases presented with the characteristic clinical and hematological profiles diagnostic of Chediak-Higashi syndrome. Careful examination of peripheral blood film in suspected cases can facilitate early diagnosis and further evaluation. In addition, recognition of early cytopenias should alert clinicians and hematologists of an impending accelerated phase. Furthermore, bone marrow aspiration is a useful diagnostic procedure for identifying tissue hemophagocytosis in such patients.

**Conflict of Interest Statement**

The authors of this paper have no conflicts of interest, including specific financial interests, relationships, and/ or affiliations relevant to the subject matter or materials included.

## Figures and Tables

**Table 1:**
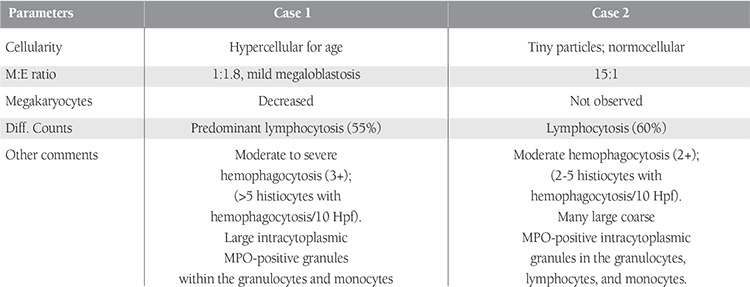
Bone marrow aspirate findings in cases 1 and 2.

**Figure 1:**
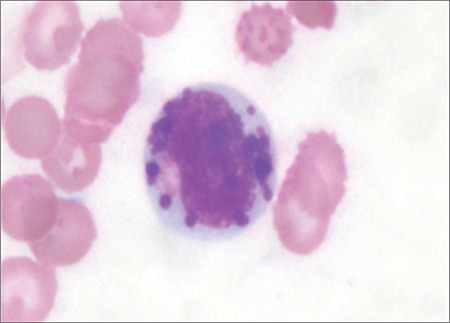
Large intracytoplasmic granules in a granulocytic cell (Giemsa stained bone marrow aspirate film; 1000×).

**Figure 2:**
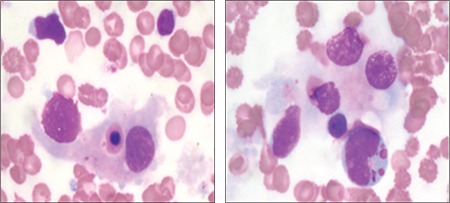
Hemophagocytosis in bone marrow smear (Giemsa stain; 1000×).
